# Vulvovaginal candidiasis among Yemeni women: prevalence of *Candida* species, biofilm formation rates, antifungal susceptibility patterns, and biofilm-associated genes *ALS1* and *HWP1*

**DOI:** 10.1186/s12866-026-05241-y

**Published:** 2026-06-16

**Authors:** Abeer Abdulmahamood Nasher, Rowa Mohammed Assayaghi, Hassan Abdulwahab Al-Shamahy, Ahmed Yahia Al-Jaufy, Arwa Mohammed Othman, Abdul-Al-Raoof Mohammad Al-Shawkany, Emad Hassan Al-Shamahi

**Affiliations:** 1 Medical Microbiology and Immunology Department, Faculty of Medicine and Health Sciences, Sana’a University, Sana’a, Yemen; 2 Molecular Genetic Department, Faculty of Veterinary Medicine, Thamar University, Thamar, Yemen; 3 Ophthalmology Department, Faculty of Medicine and Health Sciences, Sana’a University, Sana’a, Yemen

**Keywords:** *ALS1* gene, *C. albicans*, *HWP1* gene, Non-*albicans Candida*, Vulvovaginal candidiasis

## Abstract

**Background:**

Vulvovaginal candidiasis (VVC) is a common fungal infection in women, primarily caused by *Candida* species. Biofilm formation is a key virulence factor contributing to pathogenicity and antifungal resistance. This study aimed to identify *Candida* species, evaluate antifungal susceptibility, quantify biofilm formation, and detect virulence gene markers (*ALS1* and *HWP1*) in vaginal isolates from women with VVC.

**Methods:**

A cross-sectional study was conducted from December 2021 to June 2024 among 400 women attending obstetrics and gynecology clinics in Sana’a City. Vaginal swabs were collected and cultured in a microbiology laboratory. Antifungal susceptibility of isolates to nystatin, voriconazole, fluconazole, ketoconazole, clotrimazole, miconazole, itraconazole, and amphotericin B was assessed using the disk diffusion method. Biofilm formation was measured using the microtiter plate assay. The presence of *ALS1* and *HWP1* genes was determined by PCR. Data were analyzed using SPSS version 20, and associations were evaluated with the Chi-square test; *P* < .05 was considered statistically significant.

**Results:**

*C. albicans* was identified in 367 (91.8%) isolates, while non-*albicans* species accounted for 33 (8.2%). Of the 367 *C. albicans* isolates, 174 (47.4%) formed biofilms: 32 (8%) strong, 72 (18%) moderate, and 70 (17.5%) weak. Biofilm- forming isolates were associated with higher antifungal resistance, with amphotericin B (113; 64.9%) and itraconazole (108; 62.1%) showing the highest resistance, and nystatin the lowest (23; 13.2%). The *ALS1* gene was detected in all biofilm- forming *C. albicans* isolates (174; 100%), while *HWP1* was present in 82 (47.1%). Non-biofilm-forming isolates demonstrated lower resistance rates across all drugs.

**Conclusions:**

VVC remains highly prevalent in Sana’a, Yemen, with C. albicans as the dominant pathogen. These findings suggest a potential association between biofilm formation and increased antifungal resistance also the presence of key virulence genes—particularly ALS1 and HWP1. However, due to the study’s cross-sectional design, a definitive causal relationship cannot be inferred.

This study emphasizes the importance of integrating phenotypic and molecular characterization into routine diagnostics and surveillance to improve the management of resistant Candida infections. Continuous monitoring of species distribution is also warranted to detect emerging non-*albicans* species.

## Introduction

Vulvovaginal candidiasis (VVC) frequently affects young women and is associated with several risk factors, including age, diabetes, pregnancy, the use of estrogen-containing oral contraceptives, antibiotic therapy, and intrauterine devices (IUDs) [1]. Symptoms of VVC typically include erythema, burning, irritation, and vaginal discharge. *C.* albicans remains the primary causative agent of VVC, followed by various non-*albicans Candida* species [2]. While fluconazole is the primary treatment for *Candida* vaginitis, VVC is frequently diagnosed based on clinical signs and symptoms alone. However, empirical dosing and the misuse of antimycotic drugs have contributed to the evolution of varying degrees of antifungal resistance within *Candida* species [3, 4]. Indeed, the overuse of antifungal agents has led to a significant increase in drug resistance over recent decades [5, 6]. Consequently, selecting the most effective treatment for VVC requires a thorough understanding of the antifungal susceptibility profiles of *Candida* species [6].

The virulence and pathogenicity of *Candida* species are influenced by several factors, including the capacity for hyphal transition, adhesion to surfaces, the production of extracellular enzymes, and biofilm formation [7, 8]. The agglutinin-like sequence (*ALS*) gene family is the largest gene family identified in *C. albicans* and is thought to play a vital role in adhesion and biofilm formation. Genes within this family, specifically *ALS1-ALS7* and *ALS9*, are involved in the synthesis of cell surface glycoproteins that facilitate adherence to host cells [9, 10]. Notably, the *ALS1* and *ALS3* genes significantly influence attachment to host endothelial and epithelial cells [11, 12]. Additionally, the hyphal wall protein (*HWP*) encoded by the *HWP1* gene is another critical protein involved in adherence and biofilm regulation in *C. albicans* [13]. Similar to the proteins expressed by the *ALS* family, the glycosylphosphatidylinositol-linked mannoprotein *HWP1* is crucial for *Candida* adhesion [14]. Therefore, this protein is considered a significant contributor to the virulence and pathogenicity of *C. albicans* [14], with several studies indicating that *HWP1* is expressed early in the process of biofilm establishment [15, 16].

Recently, only a limited number of studies have investigated candidiasis in Yemen, spanning oral [17–19], vaginal, and urinary tract infections [20, 21]. Given the scarcity of available epidemiological data, further comprehensive research is essential to elucidate the true burden of candidiasis in the country. To address this knowledge gap, the present study was conducted to investigate vulvovaginal candidiasis (VVC) in Sana’a City, Yemen. Specifically, this investigation aimed to determine the prevalence, clinical manifestations, and species distribution of Candida associated with VVC. Furthermore, the study profiled antifungal susceptibility patterns, quantified phenotypic biofilm biomass, and determined the prevalence of the ALS1 and HWP1 virulence genes among the recovered C. albicans isolates.

## Subjects and methods

### Study design and area

The study was conducted prospectively over a 30-month period, from December 2021 to June 2024. This extended duration was intended to account for potential seasonal variation in the prevalence of vulvovaginal candidiasis (VVC) and to allow adequate recruitment of a representative sample from the target population. The study was designed as a multicenter investigation and was carried out in the obstetrics and gynecology outpatient clinics of several major hospitals in Sana’a, Yemen. The inclusion of multiple centers was aimed at improving the representativeness of the study population, as these facilities serve a wide and diverse urban female population.

All microbiological and molecular analyses were performed at the National Center for Public Health Laboratories (NCPHL) in Sana’a, Yemen. Centralization of laboratory procedures at this national reference facility ensured the strict implementation of standardized protocols, rigorous quality control measures, and access to advanced automated diagnostic systems, such as the VITEK 2 platform. Consequently, this centralized approach minimized potential inter-laboratory variability and enhanced data reproducibility.

### Study population

A total of 400 non-pregnant women presenting with clinical symptoms of vulvovaginal candidiasis (VVC) were enrolled in this cross-sectional study. The sample size was estimated based on previously reported prevalence data from a similar population in Sana’a City, Yemen [21], and was considered sufficient to describe the distribution of *Candida* species and associated virulence factors within the study population. Written informed consent was obtained from all participants after a full explanation of the study objectives. Sociodemographic information and relevant medical histories were collected using a structured questionnaire.

The study included women of reproductive age (15–55 years) who were clinically diagnosed with VVC based on the presence of vulvar pruritus, irritation, and abnormal vaginal discharge, and confirmed by laboratory isolation of *Candida* spp. Strict exclusion criteria were applied to minimize confounding factors. Women were excluded if they had cervical cancer, had received systemic or topical antifungal therapy within the previous six weeks, had used vaginal douches or spermicidal agents within 72 h prior to sampling, or were menstruating at the time of specimen collection, as these conditions may affect the vaginal microbiota and influence the accuracy of fungal isolation.

### Specimen collection and examinations

Under aseptic conditions, two sterile vaginal swabs were collected from each participant by a qualified gynecologist. One swab was placed in a sterile tube containing 2 mL of physiological saline (0.85% NaCl) for transport, while the second swab was used for direct culture [22, 23]. All samples were transported to the microbiology laboratory at the National Center for Public Health Laboratories (NCPHL), Sana’a, within one hour of collection to ensure sample viability and minimize contamination. At the laboratory, the first swab was used for direct microscopic examination using a wet mount with 10% potassium hydroxide (KOH) to detect budding yeast cells. In addition, Gram-stained smears were prepared to observe Gram-positive budding yeast cells, with or without pseudohyphae or hyphae. The second swab was inoculated onto Sabouraud dextrose agar (SDA) (HiMedia Laboratories, Mumbai, India) supplemented with chloramphenicol (50 mg/L) and gentamicin (100 mg/L) to inhibit bacterial growth [24, 25].

### Identification of *Candida* spp

Initial identification of *Candida* spp. was based on colony morphology on Sabouraud dextrose agar (SDA), where colonies typically appeared smooth, creamy-white, and yeast-like. A germ tube test was performed to differentiate *Candida albicans* from non-*albicans Candida* species. In addition, chlamydospore formation was assessed on cornmeal agar (Oxoid, UK).

For definitive species identification, all isolates were analyzed using the VITEK 2 system (bioMérieux, Marcy-l’Étoile, France) with YST ID cards (Lot No. 2432153503). Following identification, isolates were preserved in 15% glycerol and stored at − 80 °C for subsequent molecular and phenotypic analyses.

### Biofilm production by Microtiter Plate Method (MTP)

Biofilm formation was assessed using the microtiter plate (MTP) method as previously described [26, 27]. Briefly, isolates grown on Sabouraud dextrose agar (SDA) were inoculated into 2 mL of Brain-Heart Infusion (BHI) broth and incubated statically for 24 h at 37 °C in a humidified incubator. The resulting cultures were diluted 1:40 with fresh BHI broth supplemented with 2% glucose. Subsequently, 200 µL of the diluted suspension was dispensed into each well of sterile 96-well microtiter plates and incubated for an additional 24 h at 37 °C. Each isolate was tested in three independent biological replicates using separate microplates. Negative control wells contained sterile BHI broth without inoculum. The mean optical density (OD) was calculated from the three replicates for each isolate. Non-adherent cells were removed by gently washing the wells three times with phosphate-buffered saline (PBS, pH 7.2). The adherent biofilm was fixed with 2% sodium acetate for 10–15 min and stained with 0.1% (w/v) crystal violet. Excess stain was removed by washing with PBS, and the plates were air-dried for 30 min. The bound dye was then solubilized using 200 µL of 95% ethanol. Subsequently, 100 µL from each well was transferred to a new plate, and the optical density was measured at 570 nm using an ELISA plate reader (Model 680, Bio-Rad, UK). The optical density cut-off (ODc) was defined as the mean OD of negative control wells plus three standard deviations. Isolates were classified based on OD relative to ODc as follows: non-producer (OD ≤ ODc), weak producer (ODc < OD ≤ 2×ODc), moderate producer (2×ODc < OD ≤ 4×ODc), and strong producer (OD > 4×ODc) [28, 29].

### Antifungal susceptibility testing

Antifungal susceptibility testing for all *C. albicans* isolates was executed using disk diffusion method in accordance with the Clinical and Laboratory Standards Institute (CLSI) guidelines M44-A2 (Methods for Antifungal Disk Diffusion Susceptibility Testing of Yeasts; Approved Guideline—2nd ed.) [30]. Under aseptic conditions, microbial suspensions were prepared to the 0.5 McFarland standard from 24-hour colonies and inoculated onto Mueller-Hinton agar (HiMedia, India) supplemented with 2% glucose using a sterile swab. Disks supplied by HiMedia, Mumbai were placed on the agar surface. The disks used included 25 µg of fluconazole, 10 µg of ketoconazole, 100 units nystatin, 50 µg amphotericin B, 30 µg miconazole, 1 µg voriconazole, 30 µg itraconazole, and 10 µg clotrimazole. After overnight incubation, zones of inhibition (representing the antifungal effects of the drugs) were measured and evaluated. Susceptibility for fluconazole and voriconazole was interpreted according to CLSI M44 guidelines as Susceptible (S), Intermediate (I) or Resistant (R), based on zone diameter breakpoints specific to each antifungal agent and *Candida* species [26]. For ketoconazole, itraconazole, miconazole, clotrimazole, nystatin and amphotericin B, interpretation was based on manufacturer-provided interpretive criteria and quality control ranges supplied by HiMedia Laboratories [31], as no standardized CLSI disk diffusion breakpoints currently exist for these agents. Additionally, isolates exhibiting no zone of inhibition were considered non-susceptible in this study. The study was conducted as a laboratory-based surveillance investigation. The manufacturer’s interpretive charts were used as reference criteria for evaluating resistance patterns [32]. Internal quality control strains were not included in this study due to resource limitations. However, standardized inoculum density (0.5 McFarland) and incubation conditions recommended by CLSI M44 guideline were strictly followed. This approach is consistent with historical interpretations of disk diffusion results in the absence of standardized breakpoints [33] and with contemporary studies reporting similar methods when CLSI criteria are lacking [34, 35].

### Extraction of C. albicans DNA

Genomic DNA was extracted from *Candida albicans* isolates using the boiling method. Briefly, 3–5 fresh colonies grown on Sabouraud dextrose agar (SDA) were suspended in sterile distilled water and adjusted to a 0.5 McFarland standard. The suspension was transferred to sterile Eppendorf tubes and centrifuged at 10,000 rpm for 5 min. The supernatant was discarded, and the pellet was resuspended in 500 µL sterile distilled water. Samples were then incubated at 100 °C for 10 min in a heat block, followed by rapid cooling at − 80 °C for 10 min. After centrifugation at 12,000 rpm for 15 min, the supernatant containing genomic DNA was carefully collected and transferred to a new sterile tube. Extracted DNA was stored at − 80 °C until PCR analysis. DNA concentration and purity were determined using a NanoDrop spectrophotometer (Genova Nano, Jetway, UK). DNA purity was assessed using A260/A280 absorbance ratios, with values between 1.8 and 2.0 considered acceptable for downstream PCR applications [36].

### Polymerase Chain Reaction (PCR)

Conventional PCR was performed for detection of the *ALS1* and *HWP1* genes using previously published primers (Table 1). Each PCR reaction was performed in a final volume of 25 µL containing 12.5 µL of 2× PCR Master Mix, 1 µL of each primer (10 pmol), 4 µL of template DNA, and nuclease-free water to adjust the final volume.

**Table 1:** Primer sequences used for PCR amplification of *HWP1* and *ALS1* genes

Target Gene	Primer direction	Primer sequence (5′–3′)	Amplicon size (bp)	Reference
*HWP1*	Forward	ATG ACT CCA GCT GGT TC	572	[37]
	Reverse	TAG ATC AAG AAT GCA GC		
*ALS1*	Forward	GAC TAG TGA ACC AAC AAA TAC CAG A	318	[38]
	Reverse	CCA GAA GAA ACA GCA GGT GA		

### Detection of virulence gene markers by PCR

PCR amplification was carried out using a TG 40 thermal cycler (UK) under the following conditions: initial denaturation at 95 °C for 5 min; 35 cycles of denaturation at 95 °C for 30 s, annealing at 58 °C for 30 s, and extension at 72 °C for 30 s; followed by a final extension at 72 °C for 7 min. The cycling conditions were applied uniformly for both target genes. A no-template control (NTC), consisting of nuclease-free water instead of DNA template, was included in each PCR run to monitor potential contamination. The absence of amplification in the NTC confirmed that the reagents and experimental setup were free from contaminating nucleic acids. Although a positive control (reference strain) was not included due to resource limitations and the unavailability of reference strains, the identity of all isolates was confirmed prior to PCR using the VITEK 2 system. To ensure assay reliability, amplification consistency was evaluated across independent samples. Target amplicons were consistently detected at the expected fragment sizes, as verified by comparison with a DNA ladder. Electrophoretic analysis showed clear, well-defined bands without non-specific amplification or primer–dimer formation. No amplification was observed in the negative control, confirming assay specificity and indicating that the detected bands represent true target sequences.

### Gel electrophoresis

PCR products were analyzed by electrophoresis on 1.5% (w/v) agarose gel prepared in 1× TBE buffer and stained with ethidium bromide (0.5 µg/mL). Electrophoresis was performed, and DNA bands were visualized under UV transillumination. A 100 bp DNA ladder was used as a molecular size marker to determine amplicon sizes by comparison with expected fragment lengths [39].

In summary, the methodological approach of this study integrates phenotypic characterization (biofilm formation and antifungal susceptibility testing) with molecular detection of key virulence genes. The use of the VITEK 2 system ensured high accuracy in species identification, while quality control during DNA extraction guaranteed reliable PCR amplification. This comprehensive design provides a robust framework for analyzing the pathogenic potential of *Candida albicans* isolates from VVC in our region.

### Statistical analysis

Statistical analysis was performed using SPSS version 20.0 (IBM Corp., Chicago, IL, USA). Categorical variables, including the distribution of *Candida* species and the frequency of virulence genes were compared between groups using the Pearson chi-square (ꭕ^2^) test to evaluate risk factors associated with VVC. For all statistical tests, a p value < 0.05 was considered to indicate statistical significance.

## Results

Table 2 outlines the socio-demographic features of the participants (*N* = 400) across the *Candida albicans* (*n* = 367) and non-*albicans Candida* (*n* = 33) groups (overall mean age: 33.4 pm 8.4 years). The 26–35 age group was most common for *C. albicans* (48.5%), whereas the 36–45 group was most prevalent for non-*albicans* (42.4%). In secondary education predominated (45.8% and 51.5%, respectively), and the vast majority were married (97.0% each). Finally, a history of one to three pregnancies was the most frequent obstetric profile, accounting for 64.0% of *C. albicans* and 60.6% of non-*albicans* cases. Table 3 shows the distribution of *Candida* species across the study population. *Candida albicans* was overwhelmingly dominant, accounting for 91.8% (*n* = 367) of all isolates. The remaining cases consisted of rare non-*albicans* species: *Kluyveromyces marxianus*, *Nakaseomyces glabratus*, *Candida dubliniensis*, and *Candida tropicalis* each represented 1.5% (*n* = 6 each) of the total sample. Additionally, *Clavispora lusitaniae* was isolated in 1.3% (*n* = 5) of cases, while *Debaryomyces hansenii* was the least frequent at 1.0% (*n* = 4). Moreover, Table 4 displays biofilm formation capacity among the isolated *Candida* species. Biofilm production occurred exclusively within the *Candida albicans* group (43.5%, *n* = 174), categorized as moderate (18.0%, *n* = 72), weak (17.5%, *n* = 70), or strong (8.0%, *n* = 32). Conversely, 56.5% (*n* = 226) of all isolates were non-biofilm forming, which comprised 193 *C. albicans* isolates (48.25% of the total sample) and all 33 non-*albicans Candida* isolates (8.25%).

**Table 2:** Socio-demographic characteristics of women with vulvovaginal candidiasis in Sana’a, Yemen (*N* = 400)

Variables∗	Category	Cases	
		*C. albicans* (*n* = 367)	Non-albicans Candida(*n* = 33)
		No. ( % )∗	No.( % )∗
Age groups ⁄ years	15–25	63 (17.2)	4 (12.1)
	26–35	178 (48.5)	13 (39.4)
	36–45	94 (25.6)	14 (42.4)
	46–55	32 (8.7)	2 (6.1)
	Mean ± SD	33.4 ± 8.4	
Educational level	Illiterate	58 (15.8)	2 (6.1)
	Primary	56 (15.3)	6 (18.2)
	Secondary	168 (45.8)	17 (51.5)
	University	85 (23.2)	8 (24.2)
Marital status	Married	356 (97.0)	32 (97.0)
	Divorced	2 (0.5)	0 (0.0)
	Widow	9 (2.5)	1 (3.0)
Number of pregnancies	0	34 (9.3)	2 (6.1)
	1–3	235 (64.0)	20 (60.6)
	4–6	81 (22.1)	10 (30.3)
	> 6	17 (4.6)	1 (3.0)

∗ Values are presented as number (percentage). Percentages are calculated within each column

**Table 3:** Distribution of *Candida* species isolated from women with vulvovaginal candidiasis in Sana’a, Yemen (*n* = 400)

Micro-organism species*	Frequency
	No. (%)
*Candida albicans*	367 (91.8)
*Kluyveromyces marxianus (Candida spherica)∗*	6 (1.5)
*Nakaseomyces glabratus ( Candida glabrata)∗*	6 (1.5)
*Candida dubliniensis*	6 (1.5)
*Debaryomyces hansenii (Candida famata)∗*	4 (1.0)
*Candida tropicalis*	6 (1.5)
*Clavispora lusitaniae ( Candida lusitaniae)∗*	5 (1.3)
Total	400 (100)

*Taxonomic names were updated to current standards, with original names listed in parentheses [40]

**Table 4:** Biofilm formation among *Candida albicans* and non-*albicans Candida* isolates from women with vulvovaginal candidiasis (*n* = 400)

Production biofilm	Category	No. (%)
Positive (*C. albicans*) (*n* = 174)	**Total**	174 (43.5)
	Strong	32 (8.0)
	Moderate	72 (18.0)
	Weak	70 (17.5)
Non-biofilm forming isolates	*C. albicans*	193 (48.25)
	*Non-albicans Candida*	33 (8.25)
Total isolates		400 (100)

Antifungal resistance patterns among *Candida albicans* isolates shown in Table 5 which comparing biofilm-forming (*n* = 174) and non-biofilm-forming (*n* = 193) groups. Biofilm-forming isolates demonstrated significantly higher resistance rates across all eight tested antifungal agents (*p* < .001 for all comparisons). Resistance was most pronounced in the biofilm group for Amphotericin B (64.9% vs. 37.8%; χ²= 26.8) and Itraconazole (62.1% vs. 10.9%; χ²= 104.0). Substantial, statistically significant resistance gaps were also observed for Ketoconazole (39.7% vs. 7.8%), Fluconazole (35.6% vs. 7.3%), Voriconazole (33.9% vs. 6.2%), Miconazole (32.8% vs. 3.1%), and Clotrimazole (31.6% vs. 7.8%). Nystatin showed the lowest resistance overall, though it remained significantly higher among biofilm-producing isolates (13.2% vs. 3.6%; χ²= 16.2, *p* < .001). Table 6 outlines the distribution of adhesion-related virulence genes among biofilm-forming *Candida albicans* isolates (*n* = 174). The *ALS1* gene was detected in all tested isolates (100%, *n* = 174), demonstrating absolute prevalence within this cohort. In contrast, the *HWP1* gene was positive in 47.1% (*n* = 82) and negative in 52.9% (*n* = 92) of the isolates, a distribution that did not reach statistical significance (χ²= 0.575, *p* = .446). Finally, Table 7 evaluates the association between adhesion-related virulence genes and biofilm production intensity among *Candida albicans* isolates (*n* = 174). The presence of the *HWP1* gene was strongly, significantly associated with higher biofilm intensity (*p* < .001, χ²= 112.6), appearing in 100% (*n* = 32) of strong biofilm formers and 69.4% (*n* = 50) of moderate formers, but in 0% of weak biofilm formers.

**Table 5:** Antifungal resistance patterns in *C. albicans* VVC isolates stratified by biofilm-forming ability

Antifungal Agents	Biofilm forming *N* = 174	Non-biofilm forming *N* = 193	χ²	*p*
	No. (%)	No. (%)		
Nystatin	23 (13.2)	7 (3.6)	16.2	< 0.001
Voriconazole	59 (33.9)	12 (6.2)	44.8	< 0.001
Fluconazole	62 (35.6)	14 (7.3)	44.5	< 0.001
Ketoconazole	69 (39.7)	16 (7.8)	52.5	< 0.001
Clotrimazole	55 (31.6)	16 (7.8)	33.5	< 0.001
Miconazole	57 (32.8)	6 (3.1)	56.6	< 0.001
Itraconazole	108 (62.1)	21 (10.9)	104	< 0.001
Amphotericin B	113 (64.9)	73 (37.8)	26.8	< 0.001

**Table 6:** Distribution of adhesion-related virulence genes among biofilm-forming *Candida albicans* isolates (*n* = 174)

Adhesion Virulence Genes	Positive *n* (%)	Negative *n* (%)	χ²	*p*-value
*HWP1*	82 (47.1)	92 (52.9)	0.575	0.446*
*ALS1*	174 (100)	0 (0.0)	—	—

* P-value calculated by Chi-square test comparing positive vs. negative distribution

**Table 7:** Association between adhesion-related virulence genes and biofilm intensity among *Candida albicans* isolates (*n* = 174)

Virulence Gene	Strong (*n* = 32) No. (%)	Moderate (*n* = 72) No. (%)	Weak (*n* = 70) No. (%)	X^2^	*p*-value
*HWP1*	32 (100)	50 (69.4%)	0 (0%)	112.6	< 0.001
*ALS1*	32 (100)	72 (100%)	70 (100%)	—*	NS

* Chi-square could not be calculated for *ALS1* as it was constant (100% positive) across all categories

## Discussion

Vulvovaginal candidiasis (VVC) remains a common cause of vaginitis worldwide, including in Yemen [1]. However, due to the lack of mandatory reporting systems and the frequent reliance on clinical diagnosis rather than laboratory confirmation, detailed information on yeast distribution and antifungal susceptibility in Yemen is still limited. In the present study, the proportion of VVC among women presenting with microbial infections at selected clinics was 39.4%, which falls within the internationally reported range of 17% to 42% [41–43]. This rate is higher than those reported in Nigeria and India [42, 43], but lower than that reported by Rylander et al. [44]. Such variation may be explained by differences in host immune status, sociodemographic characteristics, hormonal factors, and patterns of antibiotic use [45–47].

Age and educational level appeared to influence infection patterns in this study. The highest proportion of cases was observed among women aged 26–35 years (44.5% for *C. albicans*), which is consistent with previous reports indicating a higher incidence during the reproductive years [48]. A higher proportion of cases was also observed among married women and those with secondary-level education. This may reflect differences in health awareness or healthcare-seeking behavior. Similar findings have been reported by Rathod et al. [3], while other studies have shown different patterns [49]. Among the identified species, *C. albicans* was the predominant isolate (367; 91.8%). Although *C. glabrata* is often reported as the leading non-*albicans* species in vulvovaginal candidiasis [50–53], the present study identified *C. tropicalis*, *C. glabrata*, *C. lusitaniae*, *C. dubliniensis*, and *C. famata* at relatively low frequencies (1.0–1.5%). These differences in species distribution compared with previous studies [54–56] may be attributed to geographic variation and differences in study populations.

Biofilm formation is recognized as an important virulence-related characteristic in *Candida* infections [57–59]. In the current study, 174 (43.5%) of *C. albicans* isolates demonstrated biofilm formation, including strong, moderate, and weak producers. In contrast, none of the non-*albicans Candida* isolates demonstrated detectable biofilm formation. This finding contrasts with a substantial body of literature reporting biofilm formation among several non-albicans species [60, 61]. One possible explanation is the relatively lower biofilm biomass produced by these species, which may not reach the detection threshold of the microtiter plate assay used in this study [60, 61]. In addition, differences in experimental conditions, assay sensitivity, and strain-specific variability may have contributed to this observation. The relatively small number of non-*albicans* isolates (*n* = 33; 8.2%) may have further limited the ability to detect biofilm formation. Therefore, this finding should be interpreted with caution and considered a methodological limitation rather than evidence of absence of biofilm-forming capacity. Future studies using larger sample sizes and more sensitive or complementary techniques are warranted to better characterize biofilm formation among non-albicans *Candida* species [62].

Given the established role of biofilm formation in microbial persistence and reduced therapeutic efficacy, it is biologically plausible that biofilm-forming isolates exhibit significantly elevated resistance to commonly used antifungal agents. In the present study, biofilm-producing isolates demonstrated notably high resistance rates to amphotericin B and itraconazole. However, these findings must be interpreted with caution. The disk diffusion method carries recognized technical limitations that frequently overestimate resistance to amphotericin B, primarily due to poor drug diffusion in agar media and a narrow minimum inhibitory concentration (MIC) range [63, 64]. This measurement bias is further compounded by the absence of reference quality control strains and MIC-based confirmation.

Substantial discrepancies between disk diffusion and broth microdilution methods are well-documented in the literature; for instance, Nandini and Sujatha reported an inflated resistance rate of 58.6% via disk diffusion compared to a mere 3.4% using broth microdilution [63]. Similarly, contemporary investigations in Iran and Yemen utilizing MIC-based reference methodologies documented near-universal susceptibility to amphotericin B [65–68], suggesting that the elevated resistance rates observed in this study may reflect methodological limitations of disk diffusion testing rather than true clinical resistance [30]. Conversely, the observed resistance rates for fluconazole (35.6%; *n* = 62) and voriconazole (33.9%; *n* = 59) align closely with established regional data [69–72]. These findings substantiate growing clinical concerns regarding reduced azole susceptibility among mucosal Candida isolates, a trend potentially driven by the widespread empirical administration of azole therapies and emerging selective resistance patterns [73–75].

To understand the underlying mechanisms driving these observed phenotypic traits, it is essential to evaluate the genetic architecture that governs early pathogenesis. The physical development of a robust, drug-resistant biofilm is fundamentally dictated by a complex cascade of surface adhesins and cell-wall proteins. Consequently, characterizing the distribution of core adhesion-associated virulence determinants provides critical insight into the molecular foundation sustaining both structural integrity and subsequent reduced drug penetration within Candida albicans clinical isolates [76, 77].

To this end, molecular screening of these adhesion-related virulence markers revealed that the ALS1 gene was universally present in all biofilm-forming C. albicans isolates, whereas HWP1 was identified in approximately half of the evaluated strains. The universal detection of ALS1 likely reflects its highly conserved nature across clinical C. albicans lineages or the high diagnostic sensitivity of the utilized conventional PCR assay. While both genes have been heavily implicated in the initial stages of cellular attachment and mature biofilm architecture [77]—aligning with the significant associations observed between gene carriage and biofilm intensity in this study—the frequency of HWP1 was unexpectedly lower than rates reported in systemic clinical settings. Indeed, bloodstream and urinary isolates frequently exhibit much higher HWP1 prevalence rates [78, 79], a variation that may stem from distinct anatomical isolate sources, niche-specific population characteristics, or specific methodological approaches. Critically, while PCR assays successfully confirm the presence of these virulence markers at the genomic level, they do not verify functional transcription or active gene expression during infection. Therefore, these findings represent observational genotypic associations rather than definitive evidence of a causal, mechanistic relationship, underscoring the need for future functional assays and transcriptomic analyses to fully clarify the biological significance of these virulence factors.

### Limitations

Several methodological limitations should be acknowledged. First, evaluating antifungal susceptibility for amphotericin B and itraconazole via the disk diffusion method carries inherent technical constraints that may affect resistance estimation [56, 57]. The assessment was further limited by the absence of internal reference strains and minimum inhibitory concentration (MIC)-based confirmation, potentially influencing the interpretation of susceptibility patterns. Additionally, the phenotypic microtiter plate assay may underestimate low-level biofilm biomass, particularly among non-albicans Candida species. From a molecular standpoint, conventional PCR analysis only confirmed gene presence without evaluating active expression pathways. Finally, the cross-sectional design of this study precludes causal inferences, and the sample pool was restricted to outpatient clinics in Sana’a City, which may limit the broader generalizability of the findings across Yemen.

## Conclusion

Vulvovaginal candidiasis remains a significant health concern in Sana’a, Yemen, with *C. albicans* as the predominant pathogen. A substantial proportion of isolates exhibited biofilm formation, which was significantly associated with heightened antifungal resistance. Furthermore, the presence of the *HWP1* gene strongly correlated with biofilm intensity, suggesting a functional link to genotypic virulence factors. Because biofilm formation complicates clinical management, a thorough understanding of these pathogenic mechanisms is essential. Future research utilizing transcriptomic analyses and broth microdilution reference methods is warranted to better characterize molecular resistance pathways and gene expression profiles, ultimately optimizing regional therapeutic strategies.

## Data Availability

Anyone can request access to the data from the corresponding author.

## Abbreviations

ALS: Agglutinin-like sequence
AST: Antifungal susceptibility test
BHI: Brain-heart infusion broth
CLSI: Clinical and Laboratory Standards Institute
HWP1: Hyphal Wall Protein 1
IUDs: Intrauterine devices
KOH: Potassium hydroxide
MTP: Microtiter Plate
NCPHL: The National Center for Public Health Laboratories
OD: Optical density
PCR: Polymerase Chain Reaction
SDA: Sabouraud’s dextrose agar
SPSS: Statistical Package for the Social Sciences
VVC: Vulvovaginal candidiasis
